# Failure in Medical Practice: Human Error, System Failure, or Case Severity?

**DOI:** 10.3390/healthcare10122495

**Published:** 2022-12-09

**Authors:** Mihai Dan Roman, Sorin Radu Fleacă, Adrian Gheorghe Boicean, Cosmin Ioan Mohor, Silviu Morar, Horatiu Dura, Adrian Nicolae Cristian, Dan Bratu, Ciprian Tanasescu, Adrian Teodoru, Radu Necula, Octav Russu

**Affiliations:** 1 Faculty of Medicine, University “Lucian Blaga” of Sibiu, 550024 Sibiu, Romania; 2 Faculty of Medicine, “Transilvania” University, 50036 Brasov, Romania; 3 Department for Orthopaedic Surgery, Universitatea de Medicina, Farmacie, Stiinte si Tehnologie “George Emil Palade”, 540142 Targu Mures, Romania

**Keywords:** medical failure, healthcare, system, human error, system deficiency, polytrauma, emergency, multifactorial, medical practice

## Abstract

The success rate in medical practice will probably never reach 100%. Success rates depend on many factors. Defining the success rate is both a technical and a philosophical issue. In opposition to the concept of success, medical failure should also be discussed. Its causality is multifactorial and extremely complex. Its actual rate and its real impact are unknown. In medical practice, failure depends not only on the human factor but also on the medical system and has at its center a very important variable—the patient. To combat errors, capturing, tracking, and analyzing them at an institutional level are important. Barriers such as the fear of consequences or a specific work climate or culture can affect this process. Although important data regarding medical errors and their consequences can be extracted by analyzing patient outcomes or using quality indicators, patient stories (clinical cases) seem to have the greatest impact on our subconscious as medical doctors and nurses and these may generate the corresponding and necessary reactions. Every clinical case has its own story. In this study, three different cases are presented to illustrate how human error, the limits of the system, and the particularities of the patient’s condition (severity of the disease), alone or in combination, may lead to tragic outcomes There is a need to talk openly and in a balanced way about failure, regardless of its cause, to look at things as they are, without hiding the inconvenient truth. The common goal is not to find culprits but to find solutions and create a culture of safety.

## 1. Introduction

In medical practice, both simple and extreme situations are encountered. Small details matter and can make the difference. A sub-optimal medical decision or a system defect can generate a minor imbalance or may trigger tragic outcomes.

The success rate in medical practice will never reach 100% [1]. Evaluations of medical success can be made both via technical means (scores, scales of assessment, PROMs) [2,3] and through philosophical interpretations.

In opposition to the concept of medical success, we may also consider the concept of medical failure. Failure can be defined as a deviation from desired and expected results, including both avoidable and unavoidable errors [4,5]. Its causality is multifactorial and extremely complex. It is mainly related to three factors—the human factor, referring to the healthcare provider (doctor, nurse); the medical system (infrastructure, protocols, rules, regulations, and feedback mechanisms); and the patient (the severity/specificity of their condition, and physical and psychological factors) [6].

There are mechanisms that can be used for correction and improvement. For example, successes and failures can be analyzed through the use of quality indicators such as unplanned readmissions or unplanned reoperations within 30 days. These are already used by many services. These parameters can be used to compare hospitals, clinics, or departments; to increase transparency by making them available to the public, and to ensure that patients can choose the appropriate health care. In addition, these parameters are also useful for physicians as a feedback mechanism. They can evaluate their work and educate themselves so that they can constantly improve for the benefit of the patients [7].

Feedback mechanisms should be created to avoid the future repetition of mistakes. The precise incidence of medical human/system errors (regardless of their causality) and their real impact on the final results are unknown [8]. Overload and decision-making under pressure increase the risk of error [2]. Although many errors are non-consequential, other errors can prematurely end the life of someone with a longer life expectancy [9]. Therefore, it is extremely difficult to identify and correct all the parameters/malfunctions in such complicated equations. However, it is necessary to try to properly understand the causes of errors and to be proactive in their remediation.

Medical errors are more frequent than one may think. They are the third leading cause of death in the United States after heart diseases and cancer [9]. To efficiently combat medical errors, it is of great importance to capture, track, and analyze as many medical errors as possible at the institutional level [10]. Self-reporting is the most reliable way to achieve this [11] but there are barriers, such as a poor understanding of its importance, the fear of consequences, a specific work climate or culture, the lack of feedback, the lack of a reporting system, or the time-consuming nature of the process [12]. There is also a need for specific guidelines to help clinical practitioners avoid mistakes and guide their assessment and treatment, but these are no substitute for critical thinking and rigorous learning.

Guidelines/recommendations for specific pathological conditions should help medical practitioners to avoid mistakes (and malpractice) in their clinical activities, but non-specific clinical presentations or rare conditions may lead to erroneous diagnoses and therapeutical solutions. On the other hand, adherence to the guidelines does not necessarily mean that a clinical practitioner’s conduct has been faultless [13].

If there is an absence of guidelines/recommendations, clinical practitioners should thoroughly analyze the situation, act cautiously, or ask for a second opinion if the clinical situation is complex or exceeds their capacities [14]. Although analyzing the patient outcomes (numbers) gives us some idea regarding the dimensions of the results and consequences, patient stories (clinical cases) seem to have the greatest impact on our subconscious as medical doctors and nurses and may generate the corresponding and necessary reactions.

This is because, for doctors, clinical cases—patient stories—especially those that have not evolved favorably, float like “ghosts” over our souls and come back from time to time to remind us about our limits.

Three cases are presented here to illustrate how human error, the limits of the system, and the particularities of the patient’s condition (severity of the disease), alone or in combination, may lead to tragic outcomes. These stories were experienced directly or related to the author’s medical practice and are fully relevant to the point of this thesis. Every clinical case has its own story and therefore case-based presentations have a significant impact on medical healthcare providers, encouraging them to talk about and analyze these errors.

## 2. Cases

### 2.1. First Case—Human Error/System Deficiency

The first story (case) is that of a 42-year-old male forest worker, who was active and without comorbidities. At 9:20 a.m.—at work in the woods—he suffered direct knee and proximal thigh trauma due to the fall of a large log. Because the site of the accident (a forest) was difficult to access, it took about 60 min to extract and bring him by ambulance to the ER service of a small city hospital.

**Figure 1:**
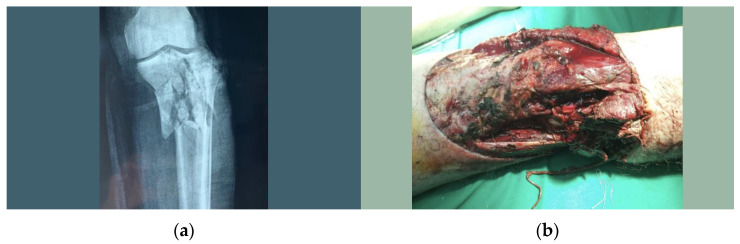
(**a**) Proximal multi-comminuted tibia fracture; (**b**) popliteal wound.

The X-rays showed a complex, multi-comminuted fracture in the proximal third of the tibia (an expression of a high-energy trauma) (Figure 1a).

Debridement and suture of the anterior superficial wound and splint immobilization were performed.

At the time of evaluation in the ER, no significant reasons to expedite its management were identified. Later, it was revealed that the clinical examination was suboptimal (vascular status examination was not properly performed), which, in the final accounting of the case, played an important role.

In their evaluation, the local medical team concluded that the fracture was too complex and exceeded the capacity of the local orthopedic surgeon and therefore decided to transfer the patient to a superior medical center. The patient arrived there at 14:00—exactly four hours and 40 min after the trauma—where the orthopedic consultation led to the identification of a missing distal pulse; sensory and motor deficit in the foot; and, upon the exploration of the posterior knee, a wound in the popliteal fossa with a potential popliteal artery injury (Figure 1b). Because at that time there was no continuous vascular surgery line in that hospital, a cardiological consultation was performed, followed by Angio-CT, which revealed the interruption of the blood flow at the level of the popliteal artery.

At 16:00 the vascular surgery consultation was performed, six hours and 30 min after the accident. The optimal timeframe (six hours) for vascular intervention was exceeded and the prognosis was extremely reserved. All this information was shared with the patient and family. Finally, surgery was performed despite the odds. Debridement, lavage, suture, and popliteal artery prosthesis were performed, and the tibial fracture was stabilized by means of an external fixator. Postoperative evolution was unfavorable and because of the vascular compromise of the leg, a thigh amputation was performed in the subsequent days.

After a seemingly favorable initial local and general postoperative evolution, the patient’s condition was aggravated due to pancreatitis associated with a sub-occlusive syndrome. Appropriate treatment was performed in the intensive care unit (ICU). Fortunately, the subsequent evolution was favorable, and the patient was discharged from intensive care after three weeks. Psychological evaluation was necessary because of depression.

The patient’s local and general evolution was favorable, a dedicated prosthesis was manufactured, and he was integrated into a recovery program. There was no legal action towards the hospital or medical caregivers.

Analyzing the present case, some positive aspects can be noted: the efficiency of the pre-hospital rescue team and the ER service provided in the second center, where the medical priorities were quickly recognized. Coherent and technically appropriate surgeries and adequate treatment in the ICU unit were performed and finally saved the patient’s life.

Unfortunately, some key factors in the decision chain finally led to the loss of the limb and a life-threatening situation.

The initial clinical exam was suboptimal—the vascular injury was not recognized. This was the main factor that decisively influenced the degree of emergency, leading to a very long waiting time in the first hospital unit, and a long duration of inter-hospital transport—using a regular ambulance instead of a helicopter. The referral of the patient to a superior hospital, but one without full-time vascular service, was directly linked to the first error.

The second factor which further aggravated the situation was system-related—the relatively long time required to assess the vascular status in the second hospital due to the deficit of permanent vascular lines and relative difficulty of accessing radiological diagnostic tools.

Therefore, both the human and system deficiencies that led to the tragic context of this patient’s story can be identified.

When human error is involved, several specific reasons can be identified: a lack of sufficient medical knowledge, a lack of interest and involvement (superficiality), burnout, personal issues (disease, psychological problems), or very busy emergency services. In an ideal setting, none of these factors should influence the outcome, but in real life, it is not always possible to have full control over them.

In cases of human error, regardless of the reasons, a feedback mechanism must be created to avoid similar situations in the future. In the development of this system, careful attention should be given to the way in which we position ourselves concerning the medical caregiver who made the error and the consequences that she/he must face. Assessments should be undertaken through root-cause analysis, rather than through a punitive system. Punishing people leads to the hiding of mistakes, instead of allowing them to be disclose and thus finding the right solution. When someone is cognitively overloaded and must constantly make decisions under pressure, there is a considerable chance of error, and this is a fact that must be taken into consideration when analyzing such a difficult situation [2]. Under these circumstances, can a human error be assimilated as a system error? Moreover, does a badly designed system generate a higher incidence of human error?

### 2.2. Second Case—Severity of Disease

The second story (case) is that of a 48-year-old garbage man, who was active and without comorbidities.

At 9:00 a.m., he suffered a direct trauma, being squeezed between a car which had skidded uncontrollably on the snow-covered road and the garbage truck. Twenty-five minutes later, the patient arrived by ambulance in the ER of a medium-sized hospital (a second-level trauma center).

**Figure 2:**
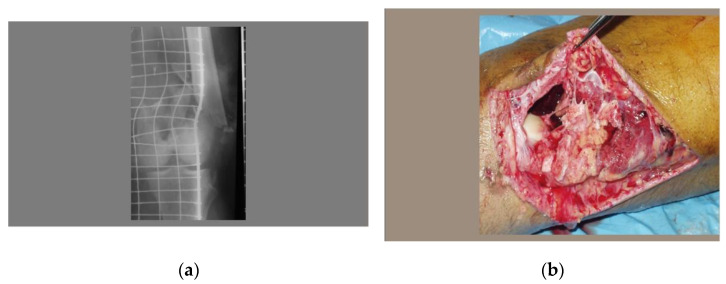
(**a**) X-ray of the supra and intercondylar multi-comminuted distal femur fracture; (**b**) open distal femur fracture—lateral view.

The initial clinical and radiological assessment revealed a grade III open comminuted fracture of the distal femur (reflecting a high-energy trauma) with popliteal artery injury (Figure 2a,b).

IV antibiotic therapy was initiated at admission and continued throughout the hospitalization. Debridement, lavage, and fixation via an external fixator were performed immediately. Because there was no vascular department in that hospital, the patient was transferred to a level 1 medical center, located one hour and forty minutes away. In the second hospital, immediate surgery was successfully performed (debridement and vascular graft of the popliteal artery). After surgery, the patient was transferred to the ICU. That took place 5 h after the initial trauma.

The initial postoperative local evolution was favorable but, unfortunately, a few days later, a septic complication occurred, compromising the vascular graft and leading eventually to thigh amputation on the seventh postoperative day. The patient was discharged about two weeks later. A dedicated prosthesis was manufactured, and he was integrated into a recovery program. There were no legal actions toward the hospital or medical caregivers.

The analysis of this case shows a well-functioning system backed up by correct human decisions. Some positive aspects may be highlighted—the rescue team’s intervention was effective; the ER service in the first clinic was also efficient, with the rapid recognition of medical priorities and adequate treatment, the surgeries were technically correct and appropriate, and collaboration and communication between the two medical centers were efficient. Unfortunately, the septic complication after vascular surgery finally compromised all these efforts. There are risk factors consistently associated with the incidence of surgical site infection (SSI) (comorbidities, advanced age, patient frailty, surgical complexity, prolonged operative time) [15], but none of these played a decisive role regarding the surgical site infection.

It is recognized that contamination of the surgical wound is an important factor in the development of SSIs [16]. In this case, the initial trauma (an open fracture) in a special septic context (a garbage truck) was the starting point. The outcome was beyond human and medical system capabilities.

This is a good example in which humans performed well and followed the guidelines and the system’s well-designed pathways. Unfortunately, the severity of the case—the infection—led to the loss of the patient’s leg.

### 2.3. Third Case

The third story (case) is that of a 35-year-old active man, without comorbidities. At 4:00 p.m., he suffered an indirect knee injury by falling from a ladder at home.

**Figure 3:**
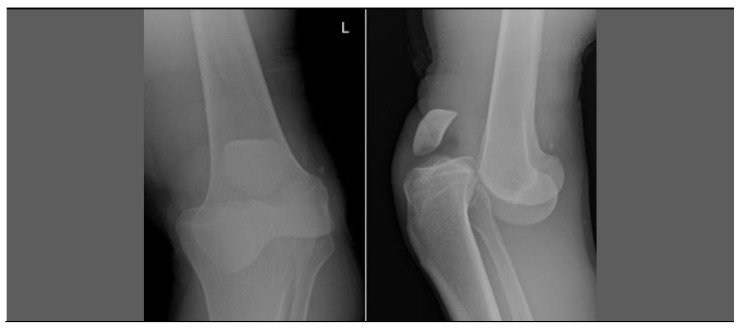
Anterior knee dislocation.

In 30 min, the patient arrived by ambulance at the ER of a medium trauma center. The clinical evaluation and the initial X-rays showed an anterior knee dislocation (Figure 3). This is a major joint trauma with multi-ligament lesions, often associated (5–43%) with vasculo-nervous injuries [17,18].

An orthopedic consultation was performed, and the dislocation was immediately reduced. Upon evaluation of the ligament status, a cold thigh and leg and the absence of a distal pulse were noticed. Forty-five minutes had passed since the onset of trauma. Because on that afternoon the surgical shift was covered by the only general surgeon who had a second specialty in vascular surgery, he was immediately called for a vascular assessment, which revealed a popliteal artery lesion. Surgery was performed immediately (thrombectomy and vascular suturing), followed by immobilization via an external fixator. The patient left the operating room about four and a half hours after the trauma. The postoperative evolution was favorable.

Analyzing this case, the same positive aspects as those mentioned before, related to both the human factor and—partially—to the system, can be noted.

On the other hand, the favorable outcome of this lucky patient can be noted: first, the orthopedic surgeon carefully evaluated and identified the vascular problem, and second, the only vascular surgeon in that hospital, who had his own necessary instruments (at that time the infrastructure of this medical center was suboptimal), was on duty. “The stars aligned themselves” to the benefit of this patient. Destiny and the human factor compensated for the deficits of the system.

## 3. Discussion

Analyzing these three cases, a similar vascular context can be identified. To efficiently help these patients, the entire (complex) network of the medical system must work properly and in coordination. In limb- and/or life-threatening situations, time may be of great importance. The human factor sometimes compensates for the system’s deficits and can change the outcome favorably. On the other hand, direct human error may have the most tragic consequences, regardless of the system, especially in specific contexts when life is at risk.

It is important that healthcare providers should be aware of the real meaning of medical errors. Poor understanding of these issues and of the importance of reporting is detrimental to the whole process of system improvement. Therefore, the process should start with the education of healthcare providers regarding what constitutes medical errors, the importance of reporting medical errors, and the fact that medical error reports are used to identify system deficiencies rather than individual faults [7].

Medical failure is correlated with the human factor—healthcare personnel—especially regarding decision-makers (physicians). Their judgment and skills make a great difference. The medical system in which they work also significantly influences the results. In addition, the outcomes are also dependent on the specific characteristics of the patient and the severity of the disease. There are also uncontrollable components such as destiny—chance or the lack of it.

In polytrauma patients, the system, the medical teams, and the patients are stressed to their limits. These require complex and often multidisciplinary surgical therapy, which involves working against the clock; therefore, any imperfection can lead to negative outcomes [19].

In critical situations that occur suddenly in people’s lives, psychological factors should also be investigated, especially if these situations are life-threatening. The psycho-emotional consequences, which appear suddenly in such conditions, and which negatively affect the quality of the patient’s life, may later cause suicidal behavior. In this context, we may recall the COVID-19 pandemic, which imbalanced the medical system; increased distress in both healthcare workers and patients; and generated, in association with other suicidal risk factors, negative consequences in the mental states of some patients, especially those with depression and other psychiatric pathologies [20,21,22]. Learning from these extreme experiences should improve our understanding and patient care [22]. Thus, the successful management of complex medical cases, in addition to the necessary emergency measures, should be treated by means of a multidisciplinary approach and cover all aspects of medical practice.

Although human error cannot be fully eliminated, strategies to reduce it should be elaborated by designing safer systems and mitigating its frequency, visibility, and consequences. Following principles that consider human limitations, as well as making errors more visible when they occur so that their effects can be intercepted, could reduce their frequency [10,23]. Other factors may also be involved, such as economic constraints, the financial revenue mechanism, public opinion (pressure), economic/social/cultural context, and the media. These all add complexity to this picture. All these factors must converge to improve outcomes and put the patient’s needs at the center [24,25].

In modern healthcare systems, which are linked to financial performance, the drive for productivity and the economics of the system may lead to severe safety constraints and adverse medical events [26]. As in other domains, healthcare systems underperform if they lack financial support. In modern medicine, performance is linked with using high-tech and expensive devices needed for accurate diagnosis and adequate therapy.

The portrayal of medical errors in the media is sometimes subjective and driven by emotions and therefore many press reports may have an accusatory tone, rather than a balanced attitude in search of solutions and improvement. This leads to a certain reluctance among healthcare givers and hospitals regarding transparency and can lead to the underreporting of failures. Physicians are more skeptical of reporting medical errors to state agencies and would prefer that reports be kept confidential [27]. This may be related to a fear of malpractice lawsuits or fines, license suspension, or sanctions against the hospital. On the other hand, exposure, and public shaming of the profession as a result of stories about medical errors in the news media play a key role in the adoption of innovations in error prevention [28].

However, at the same time, a control mechanism is needed. In an ideal world, humans should be able to correct system errors and conversely, the system should be designed to eliminate human error [25]. Unfortunately, this world does not exist. In the real world, the notion that a physician, no matter how well prepared, will perform without error during their entire career is an illusion for doctors, patients, and society [8]. All efforts must be made to strive for the best and to improve our medical systems and ourselves as doctors and human beings.

As human behavior and the design of the system play key roles in patient safety, healthcare systems should constantly perform risk assessment, prioritization, and mitigation regarding medical care processes [24].

Healthcare institutions should regularly evaluate their medical errors, report error rates transparently and perform root cause analysis. The conclusions should trigger behavioral and systemic changes [12]. This process is complex, takes time, and should involve not only specific institutions but the whole system.

Preventing medical failure and improving the success rate means, first and foremost, acknowledging the error, followed by the appropriate analysis and initiation of an action to correct it in the future (feedback) [29,30,31,32]. An organization must have the ability to learn from both large and small failures, not just by how it handles major, highly visible crises or accidents [31]. This approach entails nothing more than applying principles that are valid for any system. Unfortunately, medical culture generally discourages the admission of error, thereby significantly reducing the potential to learn from mistakes [4]. This serious analysis should be undertaken in an appropriate, neutral place that is detached from emotions and passions—a learning environment characterized by psychological safety. Hospitals should adapt their management of medical errors, with the main goal being to improve patient safety. By taking this approach, barriers to reporting medical errors may change and healthcare providers may become more open toward admitting, reporting, and discussing their mistakes, regardless of the prevailing culture. This process should be repeated continuously [7].

Our study has limitations. The case-based discussion has limited argumentation ability and cannot cover the broad spectrum of this topic. The intention of our paper is to stimulate discussion.

## 4. Conclusions

To combat medical errors, it is important to overcome specific barriers to capture, track, and analyze them at an institutional level and to create feedback mechanisms. Every clinical case has its own story and therefore case-based presentations have a significant impact on medical healthcare providers, encouraging them to talk about and analyze these errors.

In medical practice, one must rely on human and professional skills. For this to happen, there is a need to talk openly and in a balanced way about failure, regardless of its cause, to look at things as they are, without hiding the inconvenient truth. The common goal is not to find culprits but to find solutions to create a culture of safety in which the system that allowed the mistake to happen is changed for the better. Safety culture is correlated with better patient outcomes and standards; therefore, trust matters. This behavior should be correlated with specific quality indicators to better understand and correct all the deviations that endanger the quality and safety of the healthcare system.
